# Association between Indicators of Inequality and Weight Change following a Behavioural Weight Loss Intervention

**DOI:** 10.1159/000528135

**Published:** 2022-12-15

**Authors:** Jack M. Birch, Julia Mueller, Stephen J. Sharp, Simon J. Griffin, Michael P. Kelly, Jason C.G. Halford, Amy L. Ahern

**Affiliations:** ^a^MRC Epidemiology Unit, University of Cambridge, Cambridge, UK; ^b^Department of Public Health and Primary Care, University of Cambridge, Cambridge, UK; ^c^School of Psychology, University of Leeds, Leeds, UK

**Keywords:** Obesity, Weight loss, Inequalities, Primary care

## Abstract

**Introduction:**

Weight loss through behavioural weight management interventions can have important health benefits for people with obesity. However, to maximise the health benefits, weight loss must be maintained. Evidence suggests that behavioural weight loss interventions do not exacerbate inequalities in the short term. However, no study has yet considered whether inequalities exist in long-term weight change following intervention. We aimed to investigate if there are inequalities in weight change following weight loss intervention.

**Methods:**

We conducted a cohort analysis of data from the Weight Loss Referrals for Adults in Primary Care (WRAP) trial (*N* = 1,267). WRAP randomised participants to receive a brief intervention information booklet or vouchers for 12-weeks or 52-weeks of WW (formerly WeightWatchers) and followed them for 5 years. Multiple linear regression estimated the association between exposures (indicators of inequality) and outcomes (change in weight between 1- and 5-years). Each model was adjusted for the intervention group, baseline weight, weight change between baseline and 1-year, research centre, and source of the 5-year weight data.

**Results:**

Of the 1,267 participants in WRAP, 708 had weight change data available. Mean weight change between 1- and 5-years was +3.30 kg (SD 9.10 kg). A 1 year difference in age at baseline was associated with weight change of 0.11 kg ((95% CI 0.06, 0.16), *p* < 0.001). We did not find evidence of associations between ethnicity, gender, education, indices of multiple deprivation, household income, or other family members participating in a weight loss programme and weight change.

**Conclusion:**

Except for age, we did not find evidence of inequalities in weight change following a behavioural intervention. Findings further support the use of behavioural weight management interventions as part of a systems-wide approach to improving population health.

## Introduction

### Background and Rationale

Overweight and obesity are associated with an increased risk of several non-communicable diseases such as type 2 diabetes, cardiovascular disease, and some cancers (for example, bowel and post-menopausal breast cancers) [1, 2]. People living with overweight or obesity also experience higher rates of premature all-cause mortality compared to those within a healthy weight range [3]. In England in 2019, it is estimated that 68% of men and 60% of women live with overweight or obesity [4].

Inequalities in health outcomes and processes (such as intervention uptake and adherence) are known to occur across many measures summarised by the PROGRESS-Plus criteria − place of residence, race/ethnicity, occupation, gender/sex, education, socioeconomic status (SES), social capital, plus other factors for which discrimination could occur such as age and sexual orientation [5]. Inequalities are known to exist in the prevalence of overweight and obesity, such as by education and SES (those who have received fewer years of education or are more deprived are more likely to live with overweight or obesity) [6, 7]. These inequalities in obesity were particularly highlighted by the COVID-19 pandemic, where having overweight or obesity was associated with an increased risk of hospitalisation and mortality [8]. A common, effective intervention to help manage overweight and obesity is behavioural weight management [9], often following a referral from a general practitioner. Weight loss through behavioural weight management interventions can have important health benefits for people with obesity and can be cost-effective if weight loss is maintained over the long term [10]. Due to the high level of personal agency required for behavioural weight management interventions to be effective (i.e., commitment of personal resource, such as time), it is suggested that such interventions may exacerbate health inequalities by being less effective in more disadvantaged groups [11, 12].

We have previously conducted a systematic review that considered inequalities in the uptake of, adherence to, and effectiveness of behavioural weight management interventions [13]. The review noted that most trials did not find evidence of inequalities. Where inequalities were observed, trial uptake, intervention adherence, and trial attrition generally those considered “more advantaged” were favoured. Findings were more mixed for weight loss outcomes. Few trials of behavioural weight management interventions have followed participants up for more than 12 months. It is important to consider long-term weight change post-intervention as the maintenance of weight loss sustains the positive health effects associated with initial clinically significant weight loss and improves cost-effectiveness [10]. However, given that very few trials have long-term follow-up of participant weight, there is a lack of evidence on associations between characteristics of inequality (such as those outlined in the PROGRESS-Plus framework) and long-term maintenance of attained weight loss (i.e., at 5-year follow-up). Consequently, it is not known if particular sociodemographic groups demonstrate different patterns of weight change in the period following a weight loss intervention. This is important to understand as certain sociodemographic groups may benefit from additional support to maintain weight loss achieved through a behavioural intervention.

The Weight Loss Referrals for Adults in Primary Care (WRAP) trial has completed follow-up data collection at the 5-year time point, allowing for a rare opportunity to study inequalities in weight change following weight loss intervention over an extended time period. All participants in the WRAP trial received a behavioural weight loss intervention (brief intervention information booklet or vouchers for 12- or 52-weeks of WW (formerly WeightWatchers)). Previous analyses of this trial have found that trial uptake (the number of invited vs. recruited participants) was higher in older participants, people from less deprived areas, and women (although the proportion of males in WRAP was much higher than seen in similar trials or routine primary care referral) [14]; intervention attendance was higher in older participants, but there was no evidence of inequalities in attendance by any other PROGRESS-Plus criteria [15]; and there was no evidence to suggest that the greater weight loss outcomes observed in the WW groups were affected by gender, education, or income level [16]. In this current study, we investigated if there were inequalities in weight change following participation in a weight loss intervention by analysing data from the WRAP trial as a cohort.

## Methods

This study analysed data from the Weight Loss Referrals for Adults in Primary Care (WRAP) trial as a cohort.

### The WRAP Trial

The WRAP trial is a three-group randomised controlled trial of behavioural weight loss interventions. Full information about the trial design has been published elsewhere [17]. Briefly, participants were recruited through their general practice and randomised to a brief intervention, 12-week commercial weight loss programme, or a 52-week commercial weight loss programme. WRAP was registered with Current Controlled Trials on October 15, 2012 (trial registration: ISRCTN82857232). Ethical approval for WRAP was received from the NRES Committee East of England Cambridge East and local approvals from the NRES Committee North West Liverpool Central and the NRES Committee South Central Oxford. The WRAP trial was registered with Current Controlled Trials (ISRCTN82857232).

### Participants

Participants were required to be ≥18-years-old, residing in the UK, and have a BMI of ≥28 kg/m^2^. Participants were not eligible if they were pregnant or had planned pregnancy in the subsequent two years; had previous or planned bariatric surgery; were following a weight loss programme; did not speak English or had additional communication needs that would preclude them from understanding the study requirements and materials; or the participant's general practitioner considered them ineligible for inclusion (such as history of eating disorders or severe/terminal illness).

### Interventions

Participants in the behavioural programmes were given vouchers and asked to attend local WW weekly meetings and access WW web tools at no cost for the duration of the intervention (12-weeks or 52-weeks). Participants allocated to the brief intervention were given a 32-page booklet from the British Heart Foundation that comprised advice and strategies on how to lose weight. Research staff read a scripted introduction that drew attention to each section of the booklet. There were no restrictions on participants in any group accessing other weight management interventions during follow-up.

### Outcomes

Participants completed outcome assessments at baseline, 3-months, 1-year, 2-years, and 5-years. The primary outcome for this analysis was change in weight between 1-year and 5-years.

Weight measurements were made at participants' primary care practice or at the research centre by trained clinical or research staff, in line with standard operating procedures and with informed consent. Participants also reported their self-measured weight, and we collected weight data from primary care records. At the 5-year time point, if clinic-measured weight data were unavailable, the self-reported weight or weight from GP records was used (*N* = 239). Participant demographics were collected via self-report questionnaire at the baseline assessment.

The exposure variables considered for possible association with change in weight between 1- and 5-years were: (1) ethnic group *(white/ethnic minorities (excluding white minorities))*; (2) employment status *(employed/self-employed/unemployed/student/retired/unable to work/other (carer, home-maker, voluntary work))*; (3) sex *(female/male)*; (4) level of education attained *(university degree or equivalent, or higher/post-secondary education/A-levels or equivalent/GCSEs or equivalent/no formal qualifications attained)*; (5) indices of multiple deprivation (IMD) quintile *(1 (most deprived)/2/3/4/5 (least deprived))*; (6) household income *(<£20,000/£20,000 to £39,999/>£40,000)*; (7) member of household participating in a weight loss programme *(yes/no)*; and (8) age *(years)*.

### Statistical Analysis

We analysed data from the WRAP trial as a cohort rather than consider intervention versus control arms separately to estimate intervention effects. We conducted data analyses using Stata v16 (StataCorp. 2019, *Stata Statistical Software: Release 16*. StataCorp LLC., College Station, TX, USA). Mean (standard deviation) weight change was calculated within each exposure category. We defined weight maintenance as having a weight change of 3% or less from the 1-year time point [18]. We used multiple linear regression to estimate the association between each exposure and weight change from 1- to 5-years. Each model was adjusted for the intervention group, baseline weight, weight change between baseline and 1-year, research centre, and source of the 5-year weight data. A complete case analysis was performed.

#### Sensitivity Analyses

To investigate the impact of missing data on the findings, we performed a sensitivity analysis using Multiple Imputation by Chained Equations (MICE). MICE assumes that data are missing at random conditional on observed participant characteristics. Variables with ≥5% and <25% missing data had missing values imputed. The number of imputations was set to be the same as the percentage of missing data.

We conducted a further sensitivity analysis to consider if the source of the 5-year weight measurement had an impact on the results. The analysis excluded participants whose weight at the 5-year time point was collected through GP records or self-reported information. The final sensitivity analysis, added following peer review feedback, was conducting a single regression model containing all PROGRESS-Plus inequality characteristic variables included in this study to account for potential confounding between them.

## Results

### Participant Characteristics

A total of 1,267 participants were randomised to one of the three groups (211 brief intervention, 528 12-week, 528 52-week intervention). The majority of the recruited participants were women (67.8%) and of white ethnicity (89.7%). Weight data were available for 823 participants at the 1-year follow-up. At 5-year follow-up, weight data were available for 871 participants in total. Study-measured weight was available for 632 participants; weight data were extracted from GP records for 146 participants (11.5%) and collected by self-report from 93 participants (7.3%). No weight values were available for 396 (31.3%) of participants at the 5-year follow-up.

Data were available at the 1- and 5-year follow-ups for 708 participants (55.5%, Table 1). The participants included in the analyses were more likely to be retired (35.3% vs. 21.9% not included), have a university degree (41.6% vs. 31.5%), and be older (mean age at baseline of participants included in analysis was 55.7-years-old vs. 50.0-years-old for those not included). Mean weight change between 1 and 5 years was 3.30 kg (SD 9.10 kg). Mean weight change for each exposure category is presented in Table 2. Given the overall mean weight change and standard deviation values, any negative coefficients gained from the regression models (Table 3) would suggest less weight regain, or greater weight loss, in that category. Where complete data were available, weight at the 1-year time point was maintained at the 5-year time point by 28.0% of participants, >3% weight loss occurred in 16.5% of participants, and >3% weight gain occurred in 55.5% of participants (online suppl. Fig. S1; for all online suppl. material, see www.karger.com/doi/10.1159/000528135).

### Association between PROGRESS-Plus Criteria and Weight Change from 1- to 5-Years

Being 1-year older compared to other participants at baseline was associated with experiencing 0.11 kg less weight regain, or greater weight loss ((95% CI: 0.06, 0.16), *p* < 0.001). When considering occupation as the independent variable, being retired was associated with a 1.67 kg lower weight regain compared to being employed ((95% CI: 0.27, 3.08), *p* = 0.020, Table 3). Subsequently, we performed a post-hoc analysis of occupation as the independent variable controlling for age, given the differences in age distribution between occupational categories. In this analysis, being retired was no longer associated with weight change between 1- and 5-year follow-up (coefficient 1.15 (95% CI: −0.79, 3.09), *p* = 0.246; full results presented in online suppl. Table S2). No other category of occupation, when compared to being employed, was associated with weight change between 1- and 5-years in either analysis. There was no evidence of association between ethnicity, gender, education, IMD, household income, and other family members participating in a weight loss programme and weight change between 1- and 5-years.

### Sensitivity Analyses

Only one variable, education, met the conditions for performing MICE to impute missing data (between 5% and 25% missing data). The observed results using 11 imputed datasets were comparable to the primary analysis, and no associations were identified (online suppl. Table S3).

In the sensitivity analysis using only study-measured weight at the 5-year follow-up (online suppl. Table S1), being older at baseline remained associated with lower weight regain or greater weight loss, but the effect size was smaller (coefficient −0.092 (95% CI: −0.15, −0.04), *p* = 0.001). Being retired, compared to being employed, was not associated with weight change when we included only study-measured weight, neither when not controlling for age (coefficient −0.95 (95% CI: −2.48, 0.59), *p* = 0.226) nor when controlling for age (coefficient 1.66 (95% CI: −0.46, 3.78), *p* = 0.125).

The final sensitivity analysis was of all independent variables included in a single model, which also adjusted for the intervention group, baseline weight, weight change between baseline and 1-year, research centre, and source of the 5-year weight data (full results available in online suppl. Table S4). In this model, older age at baseline remained associated with lower weight regain or greater weight loss (coefficient −0.15 (95% CI: −0.25, −0.04), *p* = 0.008). The remaining results from this model were broadly consistent with our primary analyses, with the exception that being male was associated with greater weight regain or lower weight loss (coefficient 2.05 (95% CI: 0.02, 4.08), *p* = 0.048) and being unable to work (compared to being employed) was associated with lower weight regain or greater weight loss (coefficient −5.59 (−10.86, −0.32), *p* = 0.038).

## Discussion

In this study, we explored inequalities in weight change following participation in a weight loss intervention using data from the WRAP trial. Given that 55.5% of participants regained weight between the 1- and 5-year follow-up time points, the coefficients produced from the regression analyses were interpreted as indicating either less weight regain or greater weight loss compared to the reference group. We found that age at baseline was correlated with weight change between 1- and 5-years, showing that older participants experienced less weight change, and this effect was consistent across models that only used study-measured data and a model controlling for all other PROGRESS-Plus characteristics. No association was observed in our primary analysis between weight change between 1- and 5-years and other PROGRESS-Plus characteristics (ethnicity, occupation, sex, education, IMD, household income) included in our study, although in a sensitivity analysis controlling for all PROGRESS-Plus characteristics, being male was associated with greater weight regain or lesser weight loss.

### Comparison with Existing Literature

Inequalities have previously been considered in trial participation and intervention uptake, intervention adherence, and at 1-year follow-up both in the WRAP trial and other UK-based trials of behavioural weight management interventions [13]. Our study is the first to consider inequalities in weight in the longer term (i.e., at the 5-year time point).

In the previous studies that considered differential weight outcomes at 12 months, most found no association between SES or gender and weight change outcome [13], mirroring our findings of no association between these factors and weight change between 1- and 5-year follow-up. One study did identify SES as a moderator of the intervention effect [19]; however, in one intervention group, more weight was lost in those who were less deprived, and in the other intervention group, more weight was lost in those who were more deprived, showing an inconsistent relationship between SES and intervention effect. For age, one study at the 12-month time point found that older participants lost more weight [20], supporting our finding that older people regained less weight between 1- and 5-year follow-up. However, the other study that considered this issue did not find an association [21]. This lack of observed inequalities in UK-based trials could indicate that behavioural weight management interventions are similarly effective across sociodemographic groups. However, cautious interpretation is necessary, as most of these studies were not designed to identify whether inequalities in weight outcome following intervention exist and may not have been designed to detect differences between subgroups. Future research could synthesise data on weight outcomes following intervention across multiple studies, leading to more robust conclusions.

There may be several reasons why older participants regained less weight than younger participants. First, in WRAP, older participants tended to have better attendance at intervention sessions [15]. As higher levels of attendance are more likely to lead to clinically significant weight loss [22, 23], this increased level of attendance may lead to long-lasting effects of the intervention. Second, healthcare behaviour patterns of older people are generally different from those of younger people. Older adults experience fewer barriers in accessing primary care [24], meaning they are more likely to have regular consultation with healthcare professionals and may have greater healthcare need to focus on behaviours that could affect their weight. Further, specifically in terms of weight management, older people are more likely to be offered access to a weight management intervention in routine practice [25]. Third, biological factors related to ageing could affect long-term weight loss maintenance following participation in a weight loss trial. As participants reach an age of 65 years and older, a reduction in appetite and an increased rate of loss of muscle tissue have been observed [26, 27]. This may partly explain the lesser weight regain in older participants in the WRAP trial, especially as the mean age at baseline was 55.7 years old; at the 5-year follow-up, the average participant age is in the sixties. Finally, factors associated with being an older person may make it easier to attend or maintain behaviours associated with maintaining weight loss. For example, older adults may be less likely to be currently raising children or have a full-time job.

### Strengths and Limitations

This study is the first to consider if there are inequalities in weight loss maintenance following participation in a weight loss trial at the 5-year follow-up time point. The demographics of the WRAP trial sample are similar to those of the UK population in terms of SES and ethnicity [15], whereas the demographic makeup of research trials is often more affluent than that of the population.

A limitation of our study is the large amount of missing data for our primary outcome; complete outcome data were available for 55.5% of the total sample [28]. This level of missing data is common in trials of weight management interventions − the estimated retained rate in these trials at the 1-year time point was 63%, and it was 65% in WRAP [16]. Despite this, those with and without missing data were similar, indicating the sample was unlikely to have been biased by the missing data. Furthermore, our sensitivity analyses conducted to test the robustness of the data were consistent with the findings of our primary analyses. A further limitation of this study is the homogeneity of the sample, especially regarding ethnicity. The majority (94%) of the participants were self-described as white British, which limited the extent to which we could explore inequalities in weight change by ethnicity. This is reflective of the issue of diversity in clinical trials; those of ethnicities other than white are typically underrepresented [29, 30, 31]. The sensitivity analyses conducted broadly supported the results of our primary analyses, with the exception that when all PROGRESS-Plus inequality characteristics included in the study were controlled for, associations between gender and a category of occupation (unable to work) were identified.

### Implications

Despite obesity being socially patterned, with the exception of age, we did not find evidence of inequalities in weight change following weight loss intervention. Younger participants of behavioural weight management interventions may need additional support when maintaining weight loss following intervention. However, overall, our findings support the continued use of behavioural weight management interventions as part of a system-wide approach to reducing obesity and related diseases without widening existing health inequalities. Such an approach would also include population-level interventions that could support all people with obesity in maintaining weight after treatment.

## Conclusion

Except for age, we did not find evidence of inequalities in weight change following a behavioural intervention, demonstrating that behavioural weight management interventions are unlikely to generate inequalities in weight change following intervention.

## Statement of Ethics

Ethical approval for up to 2-year post-randomisation assessment was received by East of England Cambridge East and local approvals from the NRES Committee North West Liverpool Central and the NRES Committee South Central Oxford. Ethical approval for 5-years post-randomisation assessment was received from West Midlands-Coventry and Warwickshire Research Ethics Committee on December 8, 2017. The original trial (ISRCTN82857232) and 5-year follow-up (ISRCTN64986150) were prospectively registered with Current Controlled Trials on October 15, 2012, and February 01, 2018 (https://doi.org/10.1186/ISRCTN82857232; https://doi.org/10.1186/ISRCTN64986150). All participants provided written informed consent for their participation in the trial.

## Conflict of Interest Statement

WRAP was a publicly funded, independent, investigator-led trial, in which WW provided the intervention at no cost and provided funds for blood sampling and analysis for the first 2-years via an MRC Industrial Collaboration Award. Neither the funders nor WW had any role in the study design, data collection, data analysis, data interpretation, or writing of the report. Amy L. Ahern is principal investigator on two publicly funded (NIHR, MRC) trials where the intervention is provided by WW at no cost. Simon J. Griffin is principal investigator on a publicly funded (NIHR) trial in which the intervention is provided by WW at no cost. Julia Mueller is a trustee for the Association for the Study of Obesity (unpaid role). Michael P. Kelly has undertaken consultancy for Slimming World and led the obesity and weight management guidelines development for NICE from 2005 until 2014. Jason C.G. Halford has undertaken consultancy from Dupont/iFF, Mars, and Novo Nordisk (all monies paid to the University of Leeds).

## Funding Sources

Five-year follow-up of the WRAP trial was funded by the National Institute for Health Research (NIHR) under its Programme Grants for Applied Research Programme (RP-PG-0216-20010). The WRAP trial was funded by the National Prevention Research Initiative through research grant MR/J000493. Jack M. Birch, Amy L. Ahern, Simon J. Griffin, and Stephen J. Sharp are supported by the Medical Research Council (MRC) (Grant MC_UU_00006/6). The University of Cambridge has received salary support in respect of Simon J Griffin from the National Health Service in the East of England through the Clinical Academic Reserve. This work is funded by UKRI grant MC_UU_00006/6. For the purpose of open access, the author has applied a Creative Commons Attribution (CC BY) licence to any author-accepted manuscript version arising. Jason C.G. Halford is currently supported by research funding from Horizon 2020 and the American Beverage Association via the University of Liverpool.

## Author Contributions

Jack M. Birch planned and designed the study, wrote the statistical analysis plan, conducted the analysis, and led the writing and development of the final manuscript. Julia Mueller and Stephen J. Sharp planned and designed the study, provided statistical input, reviewed the statistical analysis plan, and reviewed and edited the final manuscript. Simon J. Griffin and Amy L. Ahern planned and designed the study, reviewed the statistical analysis plan, and reviewed and edited the final manuscript. Michael P. Kelly and Jason C.G. Halford planned and designed the study and reviewed and edited the final manuscript.

## Data Availability Statement

The data cannot be made publicly available because of ethical and legal considerations. Non-identifiable data and code can be made available to bona fide researchers on submission of a reasonable request to datasharing@mrc-epid.cam.ac.uk. Further enquiries can be directed to the corresponding author.

## Supplementary Material

Supplementary data

## Figures and Tables

**Table 1:** Sample characteristics by availability of data to calculate weight change

		Complete weight data available, *n* (%)	Complete weight data not available and excluded from analysis, *n* (%)	X^2^ test for association (*p* value)
Total participants		708 (55.5)	559 (44.5)	

Ethnicity	White	650 (95.2)	486 (92.6)	3.57 (0.059)
	
	Ethnic minorities (excluding white minorities)	33 (4.8)	39 (7.4)	
Occupation	Employed	319 (45.8)	287 (53.2)	31.08 (<0.001)
	
	Self-employed	63 (9.1)	52 (9.6)	
	
	Unemployed	27 (3.9)	34 (6.3)	
	
	Student	5 (0.7)	10 (1.8)	
	
	Retired	246 (35.3)	118 (21.9)	
	
	Unable to work	22 (3.2)	21 (3.9)	
	
	Other (carer, home-maker, voluntary work)	14 (2.0)	17 (3.2)	

Gender	Female	471 (66.5)	388 (69.4)	1.19 (0.275)
	
	Male	237 (33.5)	171 (30.6)	

Education	University degree or equivalent, or higher	267 (41.6)	156 (31.5)	16.91 (0.002)
	
	Post-secondary education	20 (3.1)	14 (2.8)	
	
	A-levels or equivalent	147 (22.9)	111 (22.4)	
	
	GCSEs or equivalent	181 (28.2)	182 (36.8)	
	
	No formal qualifications attained	27 (4.2)	32 (6.5)	

IMD	1 (most deprived)	72 (10.2)	83 (14.8)	11.40 (0.022)
	
	2	88 (12.4)	86 (15.4)	
	
	3	150 (21.2)	117 (20.9)	
	
	4	190 (26.8)	135 (24.2)	
	
	5 (least deprived)	208 (29.4)	136 (24.3)	

Household income	<£20,000	174 (31.2)	154 (37.4)	3.45 (0.178)
	
	£20,000 to £39,999	195 (35.6)	130 (31.6)	
	
	≥£40,000	178 (32.5)	128 (31.1)	

Other family members participating in weight loss	Yes	25 (5.0)	17 (4.8)	0.03 (0.874)
	
	No	475 (95.0)	340 (95.2)	

Age at baseline	Mean years (SD)	55.7 (12.5)	50.0 (14.4)	Logistic regression (<0.001)

Intervention group	Brief intervention	107 (15.1)	104 (18.6)	2.93 (0.231)
	
	12-week	297 (41.9)	231 (41.3)	
	
	52-week	304 (42.9)	224 (40.1)	

**Table 2:** Mean (SD) weight change by each exposure category

Exposure characteristic (number of observations)	Category	Mean weight change in kg (SD)
Ethnicity (*n* = 683)	White	3.31 (9.24)
	
	Ethnic minorities (excluding white minorities)	3.48 (7.27)

Occupation (*n* = 696)	Employed	3.76 (9.77)
	
	Self-employed	3.11 (7.07)
	
	Unemployed	3.45 (9.24)
	
	Student	–3.64 (17.48)
	
	Retired	3.09 (7.45)
	
	Unable to work	2.34 (17.04)
	
	Other (carer, home-maker, voluntary work)	2.43 (7.10)

Gender (*n* = 708)	Female	3.19 (9.63)
	
	Male	3.52 (7.97)

Education (*n* = 642)	University degree or equivalent, or higher	3.43 (8.63)
	
	Post-secondary education	4.45 (7.57)
	
	A-levels or equivalent	3.84 (8.90)
	
	GCSEs or equivalent	2.62 (10.47)
	
	No formal qualifications attained	2.28 (9.25)

SES *IMD* (*n* = 708)	1 (most deprived)	4.46 (9.35)
	
	2	2.62 (7.02)
	
	3	2.87 (9.17)
	
	4	4.19 (10.27)
	
	5 (least deprived)	2.69 (8.57)

SES *household income* (*n* = 547)	<£20,000	3.05 (9.27)
	
	£20,000 to £39,999	2.54 (10.57)
	
	≥£40,000	3.58 (8.03)

Other family members participating (*n* = 500)	Yes	2.67 (8.59)
	
	No	3.32 (9.22)

Age (*n* = 708)	≤55-years-old	3.97 (10.72)
	
	>55-years-old	2.72 (7.37)

**Table 3:** Association between PROGRESS-Plus characteristics and weight change from 1-to 5-years

Exposure characteristic (number of observations)	Category	Adjusted coefficient (95% CI)	*p* value
Ethnicity (*n* = 683)	White	Ref	-
	
	Ethnic minorities (excluding white minorities)	1.19 (–1.79, 4.17)	0.434

Occupation (*n* = 696)	Employed	Ref	-
	
	Self-employed	–0.27 (–2.54, 2.00)	0.816
	
	Unemployed	–0.05 (–3.356, 3.26)	0.978
	
	Student	–5.33 (–12.75, 2.08)	0.158
	
	Retired	–1.67 (–3.08, –0.27)	0.020
	
	Unable to work	–0.67 (–4.32, 2.99)	0.720
	
	Other (carer, home-maker, voluntary work)	–4.09 (–8.62, 0.43)	0.076

Gender (*n* = 708)	Female	Ref	–
	
	Male	1.15 (–0.24, 2.54)	0.103

Education (*n* = 642)	University degree or equivalent, or higher	Ref	–
	
	Post-secondary education	2.41 (–1.46, 6.28)	0.221
	
	A-levels or equivalent	0.81 (–0.92, 2.53)	0.358
	
	GCSEs or equivalent	–0.65 (–2.27, 0.97)	0.432
	
	No formal qualifications attained	–2.20 (–5.58, 1.18)	0.202

SES *IMD* (*n* = 708)	1 (most deprived)	Ref	–
	
	2	–1.61 (–4.31,1.08)	0.240
	
	3	–1.30 (–3.86, 1.26)	0.319
	
	4	–0.07 (–2.83, 2.69)	0.961
	
	5 (least deprived)	–1.70 (–4.61,1.22)	0.253

SES *household income* (*n* = 547)	<£20,000	Ref	–
	
	£20,000 to £39,999	–0.50 (–2.37, 1.18)	0.509
	
	≥£40,000	0.34 (–1.51,2.19)	0.718

Other family members participating (*n* = 500)	Yes	Ref	–
	
	No	0.95 (–2.46, 4.35)	0.585
	
Age (*n* = 708)	N/A	–0.11 (–0.16, –0.06)	<0.001

A multivariable model was performed to assess the association between the exposure PROGRESS-Plus characteristic and weight change. Each model was adjusted for intervention group, baseline weight, weight change between baseline and 1 year, research centre, and source of the 5-year weight data.
