# Supplementary material for: Association between Indicators of Inequality and Weight Change following a Behavioural Weight Loss Intervention
Source: Obes Facts. 2022 Dec 15;16(2):194–203. doi: 10.1159/000528135 (PMC10028366; doi:10.1159/000528135)
Supplement: Supplementary file 1 — Supplementary data [file ofa-0016-0194-s01.docx]

Table S1: Study measured weight-only results

| **Characteristic (number of observations)** | **Category** | **Coefficient (95% CI)** | **Standard error** | **P-value** |
| --- | --- | --- | --- | --- |
| Ethnicity (n=544) | White | Ref | - | - |
|  | Ethnic minorities (excluding white minorities) | 0.16 (-3.12, 3.43) | 1.67 | 0.924 |
| Occupation (n=556) | Employed by other | Ref | - | - |
|  | Self-employed | -0.59 (-3.13, 1.94) | 1.29 | 0.646 |
|  | Unemployed | 0.51 (-3.15, 4.17) | 1.86 | 0.785 |
|  | Student | -1.83 (-11.17, 7.52) | 4.76 | 0.701 |
|  | Retired | -0.95 (-2.48, 0.59) | 0.78 | 0.226 |
|  | Unable to work | -0.07 (-4.54, 4.39) | 2.27 | 0.975 |
|  | Other (carer, home-maker, voluntary work) | -3.86 (-8.61, 0.96) | 2.44 | 0.117 |
| Sex (n=563) | Female | Ref | - | - |
|  | Male | 0.95 (-0.56, 2.47) | 0.77 | 0.218 |
| Education (n=515) | University degree or equivalent, or higher | Ref | - | - |
|  | Post-secondary education | 1.87 (-2.19, 5.94) | 2.07 | 0.366 |
|  | A-levels or equivalent | 0.75 (-1.12, 2.62) | 0.95 | 0.430 |
|  | GCSEs or equivalent | -1.18 (-2.92, 0.57) | 0.89 | 0.186 |
|  | None | -2.58 (-6.44, 1.22) | 1.97 | 0.191 |
| IMD (n=563) | 1 (most deprived) | Ref | - | - |
|  | 2 | -1.09 (-4.22, 2.04) | 1.59 | 0.494 |
|  | 3 | -1.07 (-4.03, 1.88) | 1.50 | 0.476 |
|  | 4 | -0.23 (-3.36, 2.91) | 1.59 | 0.888 |
|  | 5 (least deprived) | -0.92 (-4.24, 2.40) | 1.69 | 0.587 |
| Household income (n=440) | <£20 000 | Ref | - | - |
|  | £20 000 to £39 999 | -0.99 (-2.94, 0.96) | 0.99 | 0.319 |
|  | >£40 000 | 0.04 (-2.01, 2.08) | 1.04 | 0.971 |
| Other family members participating (n=411) | Yes | Ref | - | - |
|  | No | 1.04 (-2.41, 4.48) | 1.75 | 0.556 |
| Age (n=563) | **N/A** | **-0.09 (-0.15, -0.04)** | **0.03** | **0.001** |

Table S2: Results of analysis of association between occupation and weight change controlling for age

| **Characteristic** | **Category** | **Coefficient (95% CI)** | **Standard error** | **P-value** |
| --- | --- | --- | --- | --- |
| Occupation (n=556) | Employed by other | Ref | - | - |
|  | Self-employed | 0.20 (-2.06, 2.45) | 1.15 | 0.864 |
|  | Unemployed | -0.32 (-3.60, 2.95) | 1.67 | 0.846 |
|  | Student | -4.99 (-12.33, 2.33) | 3.73 | 0.181 |
|  | Retired | 1.15 (-0.79, 3.09) | 0.99 | 0.246 |
|  | Unable to work | -0.10 (-3.72, 3.52) | 2.28 | 0.068 |
|  | Other (carer, home-maker, voluntary work) | -4.17 (-8.64, 0.30) | 2.28 | 0.068 |

Table S3: Analyses using Multiple Imputation by Chained Equations for Education

| **Characteristic** | **Category** | **Coefficient (95% CI)** | **Standard error** | **P-value** |
| --- | --- | --- | --- | --- |
| Education (n=515) | University degree or equivalent, or higher | Ref | - | - |
|  | Post-secondary education | 1.95 (-1.99, 5.89) | 2.00 | 0.330 |
|  | A-levels or equivalent | 0.60 (-1.11, 2.30) | 0.87 | 0.494 |
|  | GCSEs or equivalent | -0.66 (-2.23, 0.92) | 0.80 | 0.414 |
|  | None | -1.81 (-5.21, 1.59) | 1.73 | 0.294 |

*Table S4 Association between PROGRESS-Plus characteristics and weight change from 1- to 5-years controlling for all other measured inequality characteristics*

| **Exposure characteristic (number of observations)** | **Category** | **Adjusted coefficient (95% CI)** | **P-value** |
| --- | --- | --- | --- |
| Ethnicity (n=683) | White | Ref | - |
|  | Ethnic minorities (excluding white minorities) | 0.63 (-3.61, 4.86) | 0.772 |
| Occupation (n=696) | Employed | Ref | - |
|  | Self-employed | -1.51 (-4.46, 1.45) | 0.317 |
|  | Unemployed | -4.78 (-10.50, 0.93) | 0.101 |
|  | Student | 5.16 (-11.90, 22.2) | 0.553 |
|  | Retired | 0.14 (-2.85, 3.14) | 0.925 |
|  | **Unable to work** | **-5.59 (-10.86, -0.32)** | **0.038** |
|  | Other (carer, home-maker, voluntary work) | -1.76 (-10.44, 6.91) | 0.690 |
| Gender (n=708) | Female | Ref | - |
|  | **Male** | **2.05 (0.02, 4.08)** | **0.048** |
| Education (n=642) | University degree or equivalent, or higher | Ref | - |
|  | Post-secondary education | 1.92 (-3.01, 6.85) | 0.445 |
|  | A-levels or equivalent | 1.26 (-1.13, 3.64) | 0.301 |
|  | GCSEs or equivalent | -0.64 (-3.05, 1.76) | 0.598 |
|  | No formal qualifications attained | -2.39 (-8.04, 3.27) | 0.407 |
| Socioeconomic status *Indices of Multiple Deprivation* (n=708) | 1 (most deprived) | Ref | - |
|  | 2 | -1.82 (-5.57, 1.93) | 0.341 |
|  | 3 | -1.92 (-5.60, 1.75) | 0.305 |
|  | 4 | -0.47 (-4.48, 3.54) | 0.818 |
|  | 5 (least deprived) | -3.37 (-7.77, 1.02) | 0.132 |
| SES *Household income* (n=547) | <£20 000 | Ref | - |
|  | £20 000 to £39 999 | -0.88 (-3.29, 1.53) | 0.473 |
|  | >£40 000 | -1.00 (-3.65, 1.64) | 0.455 |
| Other family members participating (n=500) | Yes | Ref | - |
|  | No | 3.065 (-1.88, 8.01) | 0.223 |
| Age (n=708) | **N/A** | **-0.15 (-0.25, -0.04)** | **0.008** |

*A multivariable model was performed to assess the association between the exposure PROGRESS-Plus characteristic and weight change, controlling for all other inequality characteristics plus intervention group, baseline weight, weight change between baseline and 1-year, research centre, and source of the 5-year weight data.*

*Figure S1: A histogram of weight change between 1- and 5-years follow up*
